# The Lactamase Engineering Database: a critical survey of TEM sequences in public databases

**DOI:** 10.1186/1471-2164-10-390

**Published:** 2009-08-21

**Authors:** Quan Ke Thai, Fabian Bös, Jürgen Pleiss

**Affiliations:** 1 Institute of Technical Biochemistry, University of Stuttgart, Allmandring 31, 70569 Stuttgart, Germany

## Abstract

**Background:**

TEM β-lactamases are the main cause for resistance against β-lactam antibiotics. Sequence information about TEM β-lactamases is mainly found in the NCBI peptide database and TEM mutation table at . While the TEM mutation table is manually curated by experts in the lactamase field, who guarantee reliable and consistent information, the rapidly growing sequence and annotation information from the NCBI peptide database is sometimes inconsistent. Therefore, the Lactamase Engineering Database has been developed to collect the TEM β-lactamase sequences from the NCBI peptide database and the TEM mutation table, systematically compare sequence information and naming, identify inconsistencies, and thus provide a versatile tool for reconciliation of data and for an investigation of the sequence-function relationship.

**Description:**

The LacED currently provides 2399 sequence entries and 37 structure entries. Sequence information on 150 different TEM β-lactamases was derived from the TEM mutation table which provides a unique number to each protein classified as TEM β-lactamase. 293 TEM-like proteins were found in the NCBI protein database, but only 113 TEM β-lactamase were common to both data sets. The 180 TEM β-lactamases from the NCBI protein database which have not yet been assigned to a TEM number fall in three classes: (1) 89 proteins from microbial organisms and 35 proteins from cloning or expression vectors had a new mutation profile; (2) 55 proteins had inconsistent annotation in terms of TEM assignment or reported mutation profile; (3) 39 proteins are fragments. The LacED is web accessible at  and contains multisequence alignments, structure information and reconciled annotation of TEM β-lactamases. The LacED is weekly updated and supplies all data for download.

**Conclusion:**

The Lactamase Engineering Database enables a systematic analysis of TEM β-lactamase sequence and annotation data from different data sources, and thus provides a valuable tool to identify inconsistencies in sequences from the NCBI peptide database, to detect TEM β-lactamases with a novel mutation profile, and to identify new amino acid positions at which mutations can occur.

## Background

β-lactamases (EC 3.5.2.6) are the main cause for resistance against β-lactam antibiotics [1,2], because they inactivate the antibiotics by hydrolysis of the β-lactam ring of penicillins and cephalosporins. A classification scheme which was initially proposed by Ambler [3] divides β-lactamases into four classes (A, B, C, D) based on their amino acid sequences. In this scheme, β-lactamases of classes A, C, and D are serine β-lactamases, whereas class B enzymes are metallo-β-lactamases. Class A β-lactamases include, among others, the plasmid-borne TEM, SHV, and CTX-M β-lactamases, which are most commonly found in gram negative bacteria. Enzymes in this class are characterized by an active-site serine, a molecular mass of approximately 29 KDa, and the preferential hydrolysis of penicillin. At the sequence level, the TEM and SHV β-lactamase families share an identity of 67%, while the CTX-M family is more distant (sequence identity of 40% to TEM and SHV) [4].

The TEM β-lactamases are derivatives of TEM-1 and TEM-2 [5]. The premature protein consists of 286 amino acids. The first 23 amino acids at the N-terminus form the signal sequence and are removed to yield the active enzyme [6]. TEM-1 was first reported in 1965 in Greece from an *Escherichia coli *isolate [6,7]. Within a few years after the first isolation, the plasmid-mediated TEM-1 β-lactamase was identified everywhere in the world and in many different bacteria such as *Pseudomonas aeruginosa*, *Haemophilus influenzae*, *Neisseria meningitidis*, *Salmonella enterica*, or *Shigella flexneri *[5]. The β-lactamase producing bacteria are increasing in number and cause more severe infections because of continuous mutation of their lactamase genes, many of them belonging to the TEM β-lactamase family [4,8]. Until now, 167 TEM β-lactamases have been reported in a list compiled and maintained by Jacoby and Bush [9] which is referred further in this paper as "TEM mutation table". Each TEM β-lactamase is characterized by its name and mutation profile which is the set of amino acid substitutions at positions relative to the reference sequence TEM-1. A new TEM β-lactamase which is qualified and assigned in the TEM mutation table must have arisen naturally, is fully sequenced, and harbors a new mutation profile. Amino acids in the TEM β-lactamase sequence are numbered according the scheme proposed by Ambler [10]. All TEM β-lactamases included in the TEM mutation table differ from TEM-1 by up to seven mutations. 6 pairs of TEM β-lactamases are identical (TEM-1 and TEM-98, TEM-3 and TEM-14, TEM-10 and TEM-23, TEM-30 and TEM-99, TEM-34 and TEM-97, TEM-63 and TEM-64). For 11 TEM β-lactamases, the amino acid sequences have not yet been released or were withdrawn (TEM-41, 62, 69, 100, 119, 140, 148, 153, 154, 155, and 156), and therefore were excluded from our analysis. The NCBI peptide database and the TEM mutation table are developed in different ways. While the NCBI peptide database is open for submission of new sequences without external validation, the TEM mutation table is curated by experts in the β-lactamase field. As a consequence, the number of TEM-like sequences in the NCBI peptide database is rapidly growing, but is has become difficult to analyze this data, to detect inconsistencies, and eventually to reconcile sequence and naming information. Thus, 156 well-characterized TEM β-lactamases were used as a starting point to set up a TEM β-lactamase database and to identify inconsistencies in sequences from the NCBI peptide database, to detect TEM β-lactamases with a novel mutation profile, and to identify new amino acid positions at which mutations can occur.

## Construction and content

### Construction

#### Development and construction of LacED

The LacED was set up using the generic data warehouse system DWARF [11]. The underlying data model of DWARF assists the systematic building of a family-specific protein database from various data sources and supports the analysis of relationships between protein sequence and structure.

The amino acid sequence of the TEM-1 β-lactamase from *E. coli *(GenInfo Identifier GI: 41056932) was used as seed sequence. A BLAST search [12] was performed against the NCBI protein database with an E-value threshold of 10^-120 ^and without filtering of low-complexity regions. For each individual BLAST result, the GIs of the hits were extracted and the complete database entries were downloaded as XML file from the NCBI peptide database. Subsequently, information such as sequence, source organism, position-specific annotation, and functional description was extracted and parsed into the LacED by an automated retrieval system. Amino acid sequences which differed by at least one residue from the TEM-1 seed sequence were stored in the LacED as a new protein entry. Sequences which were 100% identical were assigned to the same protein entry.

Additionally, as a further data source the information of the TEM mutation table – except for 11 withdrawn or unreleased TEM β-lactamases – was incorporated into the LacED. Using the TEM-1 sequence and the mutation profiles stored in the TEM mutation table, all TEM sequences were generated and parsed into the LacED. For BLAST results representing protein structures, monomers were extracted from the PDB database [13] and deposited as structure entries.

After an initial building of the database, all entries were manually checked, and fusion proteins containing a TEM β-lactamase were removed from the database. Protein entries originating from BLAST results close to the E-value threshold and with more than 15 substitutions with respect to TEM-1 were manually removed. This threshold was chosen because it corresponds to two times the maximum number of substitutions of currently assigned TEM β-lactamases.

The LacED is updated weekly. A script searches the NCBI protein database and retrieves new entries as described above.

#### Identification and naming of TEM β-lactamase sequences

For each protein entry in the LacED, a pairwise sequence alignment with the TEM-1 β-lactamase sequence was performed using ClustalW [14]. By comparing each residue pair of the aligned sequences, changes with respect to the TEM-1 sequence were detected, and the mutation profile of the protein was identified. Subsequently, the mutation profile was matched against those mutation profiles listed in the TEM mutation table for the already assigned TEM β-lactamase sequences.

In case of 100% identity in sequence and length to an already assigned TEM β-lactamase, the sequence was added to the respective protein entry and described by the TEM number and the corresponding mutation profile. Otherwise, a new protein entry was created and its mutation profile was stored. For protein entries with a shorter or longer sequence, the difference in length can either arise from insertions or deletions inside the protein sequence or at the N- and C-termini. Insertions and deletions inside the protein sequence were annotated as: 'A249-' denotes the deletion of residue 249 with respect to TEM-1, '-41.1R -41.2T' denotes the insertion of arginine as first and threonine as second residue after position 41. Missing N- or C-terminal residues were annotated as follows: 'N-11 C-12' denotes 11 and 12 residues missing at the N- or C-termini, respectively. Protein entries with lacking residues at the N- and C-terminus but which were identical to an already existing full-length protein entry were named 'fragment of TEM-X' with X the corresponding number of the assigned TEMs

#### Multisequence alignment and feature annotation

Amino acids in the region of the signal sequence or at the active site were annotated according to the available information from source databases. The protein sequence originating from microorganisms will be referred to in this paper by the name of the organism or by "uncultured soil bacteria", while sequences from cloning vectors or other synthetic constructs will be referred to as "artificial source". Secondary structure information was calculated using DSSP [15], stored, and used for annotation. The multisequence alignment was used to enrich annotation information and control the annotation quality. The individual amino acids in the sequence and in the alignments are numbered according to the scheme suggested by Ambler [10]

#### Reconciliation of data inconsistencies

A systematic comparison of entries of the NCBI peptide database and the TEM mutation table allows to reconcile NCBI peptide database entries which are inconsistent in their mutation profile or their name. The wrong name assignment is corrected in the LacED if the mutation profile is included in TEM mutation table. A sequence with a new mutation profile is stored in the LacED as new TEM β-lactamase, even if it has been named by the authors by a (wrong) TEM name in the NCBI peptide database. A link from the reconciled LacED entry to the original NCBI peptide database entry allows the author of the respective entry to correct an erroneous entry.

### Content

#### Data content of the LacED

In total, 2399 entries resulting from the search against the NCBI protein database were parsed into the LacED. Additionally, 156 sequences including the duplicated sequences were parsed from the TEM mutation table into the LacED, except for 11 withdrawn or unreleased TEM β-lactamases. This analysis resulted in a total of 336 distinct protein entries with a unique sequence. The assignment of the 2399 parsed sequences to the 336 proteins is not equally distributed: while 20% of all sequence entries are unique, 80% of all entries correspond to only two TEM proteins, TEM-1 and TEM-116. Of the 421 TEM-1 sequences, 164 sequences originate from microorganisms while the others are from artificial sources. Of the 1380 TEM-116 sequences, only 14 sequences originate from microorganism, while the majority of them are from artificial sources such as expression or cloning vectors and other synthesis constructs. For E-values above 10^-120^, the search against the NCBI protein database resulted in β-lactamases of the PLA, OKB-P, and LEN type, which are chromosomally encoded β-lactamases. At an E-value threshold of 10^-104^, the first SHV β-lactamases appear in the search results.

Information on TEM crystal structures was also included in the LacED. 37 PDB structures were assigned to 22 different LacED entries based on sequence identity. All 37 structures lack the 23 amino acids of the signal sequence.

**Figure 1:**
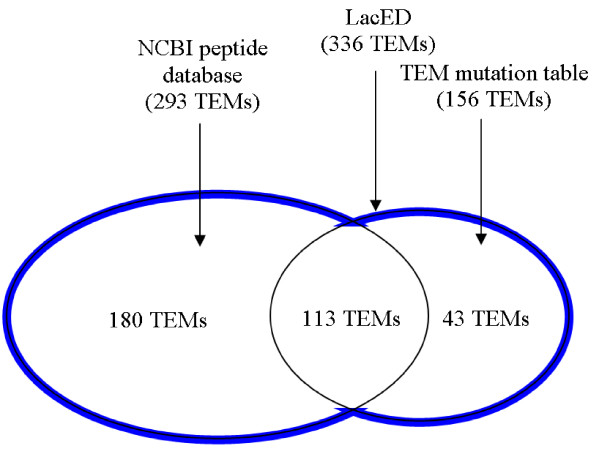
**The data sources of protein sequences in LacED**.

The majority of the 336 LacED protein entries were found in the NCBI peptide database and were not described in the TEM mutation table (180 sequences). 113 protein sequences were found in both databases and 43 sequences were characterized in the TEM mutation table but not found in the NCBI (figure 1).

#### New mutation profiles in sequences of microbial origin

Half of the 180 TEM β-lactamases with a new mutation profile which have been found in the NCBI peptide database but not described yet in the TEM mutation table were of microbial origin. Of them, 26 protein entries were full length sequences found in defined organisms (table 1), 6 protein entries were shorter sequences lacking several residues (table 2), and 57 protein entries were protein fragments extracted from "uncultured soil bacteria" (Additional file 1: Table S1).

**Table 1:** Sequences with new mutation profiles from microbial source

**GI**	**Mutation**	**Source organism**
112785110	R65H	*Escherichia coli*

112785108	T118A	*Escherichia coli*

223006551	M155I	*Proteus mirabilis*

154263799	A184V	*Escherichia sp*. Sflu5

38456216	M186I	*Streptococcus pneumoniae*

157676818	T188K	*Escherichia coli*

12018137	E240G	*Escherichia coli*

213133718	N276I	*Escherichia coli*

112785106	A280D	*Escherichia coli*

34099626	A284T	*Acinetobacter baumannii*

190410144	F24L A25S	*Neisseria gonorrhoeae*

67464385	S70G V84I A184V	*Escherichia coli*

77021517	S53G V84I A184V	*Shigella flexneri*

82395024	P226L R244L A248E	*Escherichia coli*

110610033	Q39K R164S E240K	*Proteus mirabilis*

55140586	L12A I13L P14S G92D	*Pseudomonas aeruginosa*

33318322	Q39K E104K R164S K215R	*Klebsiella pneumoniae*

112785120	V84I I173K A184V K234T	*Enterococcus gallinarum*

57236771	I5F K55G H153R	*Vibrio cholerae*

110835657	G283E A284K S285Q L286K I287C K288L H289T W290D	*Acetobacter pasteurianus*

2970345	Q39K L51P E104K R164S A187R S223C F230L	*Providencia stuartii*

15149330	S4D I5P H289L	*Escherichia coli*

15149328	S4D I5P M69V H289L	*Escherichia coli*

15149326	S4D I5P R244S H289L	*Escherichia coli*

20805894	S4D I5P V80E G196S N276S	*Salmonella typhimurium*

2648043	A42S R43T V44S P145S K146Q R178A E212- G238R	*Serratia marcescens*

**Table 2:** Protein fragments from microbial source containing new mutations

**GI**	**Number of ** **residues missing**	**Source organism**	**Description**
90110162	C-2	*Escherichia col*i	New mutation profile, the mutation at M211 is not in TEM mutation table

26245317	C-3	*Erwinia amylovora*	P145X T181X A184V I263X T265P, there are three undefined residues in the sequence. The mutation T265P is not in the TEM mutation table

166078558	C-14	Escherichia coli	N276K is not in TEM mutation table

166078576	C-13	*Escherichia col*i	The new mutation N274D is not in the TEM mutation table

117950175	N-1 C-8	*Salmonella typhimurium*	The new mutation I282E is not in the TEM mutation table

45758090	C-17	*Salmonella enterica*	New mutation profile T266D S268E Q269S A270G T271N. The mutation at position 266, 269, 270, 271 are not in TEM mutation table, at the end of the sequence.

As an example, protein entry GI 157676818 originates from *E. coli *and was directly submitted as nucleotide sequence to the EMBL Nucleotide Sequence Database. The translated sequence is of full length and has a substitution of threonine at position 188 to lysine. A substitution at this position has not yet been documented in the TEM mutation table. Protein entry GI 90110162 also originates from *E. coli*, lacks 2 C-terminal amino acids, and has the mutation profile R204W, M211V. The occurrence of substitutions at position 204 is known (e.g. R204Q in TEM-70), but a substitution to tryptophan has not been described yet. Position 211 is a novel location for a substitution in the TEM β-lactamase sequence.

### New mutation profiles in sequences from artificial sources

In total, 31 full length proteins with a new mutation profile were identified from artificial sources such as cloning or expression vectors. The amino acid substitutions occur not only at the already described positions, but also at novel positions different from those reported in the TEM mutation table. Most novel mutation profiles include the substitutions V84I and A184V, and therefore are probably derived from TEM-116.

As an example, the protein from cloning vector pRSQ (GI 1003002) has a length of 286 residues and the mutation profile V84I, L152F, A184V. The substitutions V84I and A184V are found in TEM-116, but the substitution L152F is new. A list of the 31 proteins from artificial sources, together with their mutation profile and corresponding GI is provided in Additional file 1: Table S2.

### Data inconsistencies

A set of 55 protein entries was found to have inconsistent annotation. Of these, 12 protein entries were inconsistent in terms of TEM number assignment or in terms of the reported mutation profile. They show differences between their respective entries in the NCBI peptide database and the TEM mutation table, or between the NCBI peptide database entry and the respective publication (table 3). As an example, the protein sequence with the GI 15081590 is described as TEM-102 with the mutation profile A25V, H26R, A184V, L250V, but the TEM mutation table lists TEM-102 with the completely different mutation profile L21F, R164S, T265M. While the protein sequence with the GI 15081590 was submitted to GenBank in 2002, the TEM-102 entry in the TEM mutation table was documented in 2003 [16].

**Table 3:** Inconsistencies of mutation profiles reported in different data sources

**Name and mutation profile reported by**		**Inconsistency**
	
**NCBI peptide database**	TEM mutation table*	
RTEM-1: F66S A184V (GI:2623824)	RTEM-1 = TEM-1 no mutation	Same name, different mutation profile

TEM-1: F24L A25S (GI:89112911)	TEM-1: no mutation	Same name, different mutation profile

(GI: 213133718)	TEM-1: no mutation	Same name, different mutation profile

TEM-26B beta-lactamase: R164S (GI:149169)	TEM-12: R164S	Different name, same mutation profile

IRT-18: M69L R244S (GI:6688989)	TEM-77: M69L R244S	IRT-18 is TEM-73*. Different name, same mutation profile

TEM-102: A25V H26R A184V L250V (GI:15081590)	TEM-102: L21F R164S T265M	Same name, different mutation profile

TEM-128: A284T (GI:38456216)	TEM-128: D157E	Same name, different mutation profile

M186I (GI:38456216)	Q39K E104K R164S	Same name, different mutation profile

TEM-136: L12A I13L P14S G92D (GI:55140586)	TEM-136: R164S A237T E240K S268G	Same name, different mutation profile

TEM-144: P226L R244L A248E (GI:82395024)	TEM-144: R164C E240K	Same name, different mutation profile

(GI:161367442)	TEM-162: E28K D38N E64K V84I N100S L102V A184V	Same name, different mutation profile

TEM-166: S59G R164S A237T E240K (GI:183584860)	TEM-166: R120G	Same name, different mutation profile

### Fragments of assigned TEM β-lactamases

Another group of 39 protein entries consists of TEM β-lactamase sequences with lacking residues but otherwise known mutation profile. They were identified as fragments corresponding to already assigned TEM β-lactamases. As an example, the protein entries GI 28192514 and 145558692 are annotated as TEM-117 and TEM-131, respectively. However, the sequences are missing 12 and 11 residues at the N-terminal and 19 and 12 residues at the C-terminus, respectively. A list of all 39 protein entries with missing residues is provided (Additional file 1: Table S3).

### Analysis of amino acid substitutions and substitution positions

**Figure 2:**
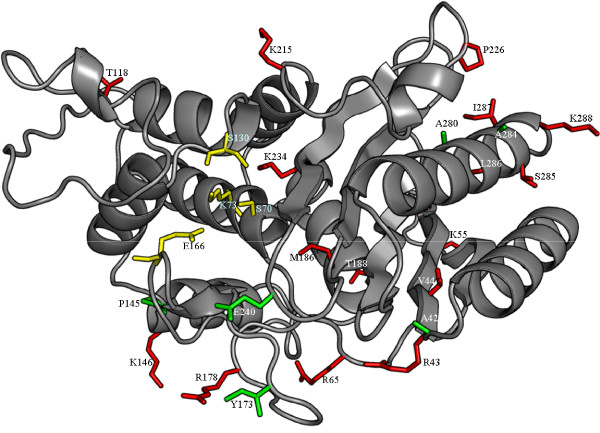
**The structure of TEM-1 β-lactamases **(PDB entry 1BTL) **with the positions of substitutions found in sequences from microbial origin**. Amino acid side chains are shown in stick representation: substitutions occurring at already described positions (green), substitutions at novel positions (red), and active site residues (yellow). Residues at positions 53, 238, 248, 283 can not be seen from this view.

Besides the known amino acid substitutions documented in the TEM mutation table, 35 new substitutions in protein entries originating from microbial organisms have been identified in full length sequences and in fragments. New substitutions occurred either at already described positions with an exchange into a new residue (10 substitutions) or at completely new positions in the protein sequence (Additional file 1: Table S4). The substitutions are spread over the complete protein sequence, including the signal peptide. Most of the substitutions are located at the protein surface and are distant from the active site, with the exception of Lys234 whose side chain is 3.4 Å distant to the Ser70 side chain and Met186 which is buried in the protein core (figure 2).

**Figure 3:**
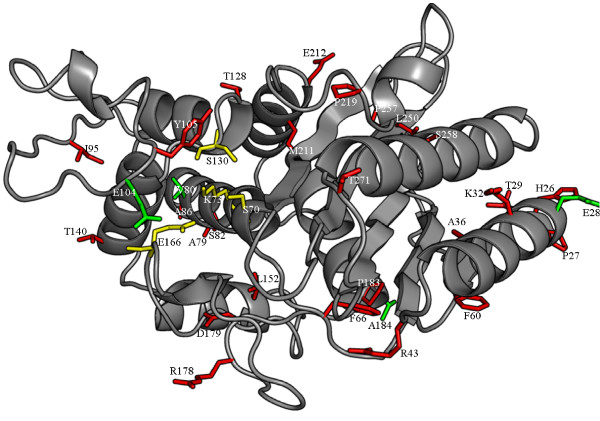
**The structure of TEM-1 β-lactamases **(PDB entry 1BTL) **with the positions of substitutions found in sequences from artificial sources**. (PDB: 1BTL). Amino acid side chains are shown in stick representation: substitutions occurring at already described positions (green), substitutions at novel positions (red), and active site residues (yellow).

Protein entries derived from artificial sources such as cloning vectors contained 31 novel substitutions, including 5 substitutions at positions which were already described for sequences originating from microbial sources (Additional file 1: Table S5). The localization of the substitutions in the protein structure is similar to those found in sequences from microbial origin (figure 3).

Comparing the novel substitutions found in sequences originating from microbial organisms with those originating from artificial sources, only 12 positions are in common (position 25, 43, 80, 84, 104, 164, 178, 184, 187, 212, 240, and 284). However, in most cases the substituted amino acids were different depending on the source of the sequence.

Additionally, 64 novel substitutions have been found in uncultured soil bacteria. Details on these substitutions are provided as additional file (Additional file 1: Table S6).

## Utility

A multisequence alignment of all 336 protein entries was generated using CLUSTALW. For protein structures, all sequence entries were included and displayed with aligned secondary structure information. Proteins were labeled by the GIs and are linked to the NCBI protein database. Annotation of individual residues is visualized by color-coding in the alignment and by moving the mouse cursor over the respective colored residues. Protein tables provide information on the protein name, mutation, number of residues missing at the N- and C-terminal (in case of fragments) as well as on the source organism. The accession codes corresponding to each LacED protein entry are also shown and linked to the source database. As an alternative to the multisequence alignment, the TEM variants are visualized as mutations relative to the sequence of TEM-1. Substitution positions are colored and annotated by the name of the respective variant (TEM-2, TEM-3...). BLAST search against the LacED can be done using the BLAST function.

## Discussion

### Differences between data sources

336 TEM β-lactamase protein sequences were retrieved from two data sources, the NCBI peptide database [17] and the TEM mutation table [9]. Only 113 of the 156 TEM β-lactamases of the TEM mutation table could be found in the NCBI peptide database. The references given in the TEM mutation table for the remaining 43 TEM β-lactamase sequences were either labeled as "personal communication" (TEM-18, 19, 61, and 163, 165), or the respective publication did not contain a database accession number.

On the other hand, the search in the NCBI peptide database resulted in 180 new TEM β-lactamase sequences as compared to the TEM mutation table, including 89 sequences from microbial origin and 91 synthetic constructs.

### Detection of novel TEM β-lactamase variants and amino acid substitution

Most of the novel mutation profiles originate from artificial constructs such as cloning vectors, and hence do not qualify for the assignment of a new TEM number. If it is assumed that the TEM β-lactamase encoded by such a construct is functional, the analysis of novel mutation profiles is valuable data, though, to study sequence-function relationships and to predict mutations which might also occur in microbial TEM β-lactamases in the future.

However, 26 full length TEM β-lactamase sequences with a novel mutation profile were detected, which originate from microbial organisms and thus satisfy the requirements of Jacoby and Bush for assignment to a new TEM number. It should be noted that the occurrence of these novel sequences in public databases does not necessarily mean that the corresponding proteins are expressed. It might well be the case that sequencing errors or errors upon data transfer are the reason for the novel mutation profile.

In addition to the full length sequences, 6 fragments from microbial source with a new mutation profile were found. Because there might be more substitutions present in the lacking part of the sequence, the current information on these fragments is not sufficient to assign a new TEM number. However, the new mutation sites or combinations of mutations contribute to the study of sequence-function relationships.

The 57 TEM β-lactamase sequences originating from uncultured soil bacteria constitute the largest number of sequences with a novel mutation profile. Because "uncultured soil bacterium" has to be considered as a natural source, these proteins would qualify for the assignment of new TEM numbers, but certainly further study of these sequences is necessary.

The systematic analysis of all sequences identified 35 novel amino acid substitutions in sequences from microbial origin, which are not yet described in the TEM mutation table. Among these mutations, P226 and K234 provide an insight into the evolution of new specificities. From a previous *in vitro *experiment with the antibiotic ampicillin, it was concluded that amino acid substitutions in these positions are not tolerated [18]. However, the authors suggested that mutations at these sites might be required for hydrolysis of other β-lactam antibiotics, which has been demonstrated by site-directed mutagenesis for position 234 [19]. The occurrence of β-lactamases from *Enterococcus gallinarum *harboring the substitution K234T (GI 112785120) and from *E. coli *harboring the substitution P226L (GI 82395024) demonstrates that *in vitro *studies can predict events in natural evolution of TEM β-lactamases, and that a challenge by different antibiotics will lead to different responses. Thus it confirms the high plasticity of β-lactamase sequences in response to evolutive pressure by a broad spectrum of antibiotics.

### Data inconsistencies and reconciliation

The 12 NCBI peptide database entries with inconsistencies include wrongly named sequences (IRT-18 and TEM-77, GI 6688989) and sequences for which the mutation profiles do not correspond to those listed in the TEM mutation table.

The protein sequence GI 38456216 was submitted as '*Streptococcus pneumoniae *plasmid beta-lactamase TEM-129' to the NCBI peptide database [20]. The translated protein sequence has the mutation profile M186I, which differs from the mutation profile reported for TEM-129 in the TEM mutation table (Q39K, E104K, and R164S). So far, this is the only case reporting that *Streptococcus pneumoniae *contains a known β-lactamases. The mistake was then identified with the presence of β-lactamase DNA in some brands of commercial *Taq *polymerase [21], but the entry is still available in the NCBI peptide database.

An interesting case is the occurrence of 5 sequences with the substitutions S4D, I5P in the leader peptide. All of them have been reported to originate from Irish bacteria strains (GI 15149330, 15149328, 15149342, 42525360, and 13676302). Three of them were directly submitted by the same authors into the public database, two sequences were submitted along with a publication [16], describing one of the sequences as TEM-102 with the mutation profile L21F, R164S, T265M. However, the substitutions S4D and I5P that we found in all five sequences were not mentioned in the publication, nor listed in the TEM mutation table.

The inconsistencies were reconciled in the LacED based on the information from the TEM mutation table. Most of the inconsistencies are either sequences with the same TEM number but a different mutation profile in the NCBI peptide database and the TEM mutation table or sequences with the same mutation profile but a different TEM number in the NCBI peptide database and the TEM mutation table. In the first case, the sequence was considered as a new TEM-like sequence, in the second case the information of this entry was corrected according to the TEM mutation table and stored in the LacED. A link from each LacED entry to the original entry in the NCBI peptide database allows the authors of the submitted sequence to trace the inconsistency and to correct the data.

#### Open questions

The observed inconsistencies in NCBI peptide database entries between the annotated TEM name and the mutation profile demonstrate the need for a careful analysis of new sequence entries by a systematic comparison to already existing sequences. Although major scientific journals require authors who submit a new TEM β-lactamase sequence to have the agreement of the curator of TEM mutation table prior to publication, the NCBI peptide database allows the submission of new sequences without further validation. A major problem of the observed inconsistencies lies in the fact that researchers who are not TEM β-lactamase experts might rely on the information found in the NCBI peptide database. We therefore consider the LacED as a valuable tool to detect such inconsistencies and report them to the interested community. Therefore, a tool such as the LacED provides a valuable approach to systematically analyze new entries and to compare them to existing sequence and annotation information.

## Conclusion

The amount of sequence and structure data of TEM β-lactamases available in public databases is increasing rapidly. The Lactamase Engineering Database enables a systematic analysis of sequence and annotation information from different data sources. It helps to identify inconsistencies and to discover novel mutation profiles. In the future, the Lactamase Engineering Database will be extended to the other lactamase families and classes.

## Availability and requirements

The LacED is accessible at  by a JavaScript-enabled WWW browser.

## Abbreviations

GI: GenInfo Identifier; LacED: Lactamase Engineering Database.

## Authors' contributions

QKT developed the database, built the web pages, analyzed the data and drafted the manuscript. FB contributed to data analysis and to writing of the manuscript. JP supervised the study and finalized the manuscript. All authors read and approved the final manuscript.

## Supplementary Material

Additional file 1 **Supplemental tables**. This file contains tables S1, S2, S3, S4, S5, S6 mentioned in the text. Click here for file
