# Supplementary material for: The Lactamase Engineering Database: a critical survey of TEM sequences in public databases
Source: BMC Genomics. 2009 Aug 21;10:390. doi: 10.1186/1471-2164-10-390 (PMC2742552; doi:10.1186/1471-2164-10-390)
Supplement: Additional file 1 — Supplemental tables. This file contains tables S1, S2, S3, S4, S5, S6 mentioned in the text. [file 1471-2164-10-390-S1.pdf]

The Lactamase Engineering Database: a critical survey of TEM sequences in public databases

Quan Ke Thai, Fabian Bös, Jürgen Pleiss[§]

Institute of Technical Biochemistry, University of Stuttgart, Allmandring 31, 70569 Stuttgart, Germany

[§]Corresponding author

Email addresses:

Quan Ke Thai: tkquan@itb.uni-stuttgart.de
Fabian Bös: Fabian.Boes@itb.uni-stuttgart.de
Jürgen Pleiss: Juergen.Pleiss@itb.uni-stuttgart.de

SUPPLEMENTARY MATERIAL

Table S1: GIs of 57 LacED protein fragments from uncultured soil bacteria

146260002, 146259995, 146259997, 146260070, 146260157, 146260176, 146260022, 146260092, 146260130, 146260119, 146260184, 146260174, 146260172, 146260232, 146260059, 146260162, 146260086, 146260127, 146260117, 146260054, 146260028, 146260209, 146260153, 146260043, 146260080, 146260192, 146260160, 146260190, 146259999, 146260105, 146260169, 146260061, 146260094, 146260199, 146260230, 146260134, 146260195, 146260017, 146260122, 146260101, 146260211, 146260007, 146260151, 146260182, 146260220, 146260186, 146260240, 146260089, 146260207, 146260068, 146260180, 146260032, 146260045, 146260227, 146260009, 146260202, 146260138

Table S2: GIs and mutation profiles of 31 LacED proteins from artificial sources

GI	MUTATION PROFILE
37778088, 37778090	V84I A86V A184V
4732115, 4928116	V84I A86P A184V
16973670, 20301817, 22476926, 69048019, 69048101, 69048159, 69048310, 69048378, 69048448, 89158284	F60L V84I T140A A184V P219S
3328184, 13488584, 34421984, 37223185, 37575402, 37575407, 37575411, 37575415, 37575419	V84I A184V T271A
18996298, 18996302, 29788131	V84I A184V M211L E212L
2293126, 2293129	V84I D179H A184V
74054892	V84I A184V A284H
74054894	V84I A184V A284L
74054890	V84I A184V D273E A284L

129278849	F66S A184V
224611742	V84I A184V Q278L
183584860	S59G R164S A237T E240K
84570489, 154936855	G78D A79T V80L V84I R178S A184V A187P P257R
1003002, 1546042, 1546047, 2801668, 6642842, 6642845, 6642849, 6642853, 37812662, 37812675, 38327509	V84I L152F A184V
4884789	V84I H96L A184V A187P
454853, 454856	P183L A184Y
2995959, 4808836, 5639950, 22774036, 27573252, 27573255, 27573258, 134105580, 145307007, 164470782, 164470785, 164470788, 164470791, 187969496, 187969501, 187969506, 187969511, 187969516, 187969521, 187969526, 187969531, 187969536, 187969541	V84I
15081590	A25V H26R A184V L250V
4732037, 22000816, 452349, 4322593, 4322640, 13094135, 13094138, 22001013	G78H A79T V80L V84I I95A H96R A184V A187P
208244	M211L
105958774	V84D
116282558, 116282561, 116282565, 116282569, 116282573, 116282577	V84I T128S L152F A184V
2589218	A184P
13492214, 13492219	G78H A79T V80L V84I I95A H96R R178S A184V A187P
55669167	R43L V84I A184V
6457305	P27F E28D T29L K32F A36C S82F V84I A184V

1840129, 3834576	E104D Y105A
4731623, 4731626, 1262901, 3135594, 3135597, 3135600, 3135603, 3135606, 3135609, 3777575, 3777578, 3777581, 9885347, 50253789, 50253793, 50253797, 50253801, 50253805	V84I A184V A187P
595783, 644776, 145307011	A184T
53987101	V84I A184V S258F
146743440, 146743444, 146743446	V84I Y105A A184V

Table S3: GIs, mutation profiles and missing residue of 39 LacED protein entries which are fragments with known mutation profile. ‘C-5’ means “this sequence lacks 5 residues at the C terminal”; ‘N-12’ means “this sequence lacks 12 residues at the N terminal”

**GI**	**Mutation profile like**	**Missing residue**
44194076, 44194080, 44194082, 44194084	TEM-1	C-5
166078564, 166078572	TEM-1	C-14
46276339	TEM-1	N-12 C-10
44194078	TEM-1	C-4
166078560	TEM-1	C-10
71273528, 166078588	TEM-1	C-17
166078552, 166078556, 166078562, 166078566, 166078568, 166078570, 166078574, 166078584	TEM-1	C-15
166078554	TEM-1	C-13
56123232	TEM-1	N-9 C-8
166078558	TEM-1	C-14
166078578, 166078582	TEM-1	C-18
166078550, 166078580, 166078590	TEM-1	C-12
166078586	TEM-1	C-22
28195099	TEM-7	N-12 C-13

484096	TEM-10	C-17
8272638	TEM-17	C-9
495299	TEM-26	C-17
27817549	TEM-42	N-3 C-2
4559300	TEM-52	C-7
5650761	TEM-59	C-9
2853027	TEM-63	N-1 C-12
23505686	TEM-75	N-12 C-13
14700044	TEM-89	C-8
28192516	TEM-110	N-12 C-13
46244158	TEM-116	N-22 C-35
46244160	TEM-116	N-22 C-32
46244152	TEM-116	N-17 C-28
46244156	TEM-116	N-24 C-28
145306442	TEM-116	C-3
156106793, 156106796, 156106801, 156106807	TEM-116	N-66
47846294	TEM-116	C-1
169401966	TEM-116	N-14
46244162	TEM-116	N-16 C-28
414523, 644827, 110227251, 110227261, 110227278, 188485338	TEM-116	N-65
19595434	TEM-116	C-14
28192514	TEM-117	N-12 C-19
23505688	TEM-118	N-12 C-13
45268493	TEM-128	C-12
145558692	TEM-131	N-11 C-12

Table S4: New mutation profiles derived from microbial organisms

Position	4*	5*	12*	13*	14*	24*	25*	39	42	43*	44*	51	53*	55*	65*	69	70*	80	84	92	104	118*	145	146*	153	155*	164	173	178*	184	186*	187*	188*	196	212*	215*	223*	226*	230*	234*	238	240	244	248*	276	280	283*	284	285*	286*	287*	288*	289	290*
GI	S	I	L	I	P	F	A	Q	A	R	V	L	S	K	R	M	S	V	V	G	E	T	P	K	H	M	R	I	R	A	M	A	T	G	E	K	S	P	F	K	G	E	R	A	N	A	G	A	S	L	I	K	H	W
112785110***															H																																							
112785108***																						A																																
223006551***																										I																												
154263799																														V																								
38456216***																															I																						
157676818***																																	K																				
12018137																																										G											
213133718																																													I**								
112785106																																														D**							
34099626																																																T**					
190410144***						L	S																																															
67464385																	G		I											V																								
77021517													G						I											V																								
82395024																																						L					L	E											
110610033								K																				S																K											
55140586			A	L	S															D																																	
33318322								K													K						S									R																			
112785120																			I									K*		V										T															
57236771		F												G											R																													
110835657																																															E	K**	Q	K	C	L	T**	D
2970345								K				P										K						S					R					C		L															
15149330	D	P																																																			L	
15149328	D	P														V																																				L	
15149326	D	P																																										S										L	
20805894	D	P																E																	S												S**									
2648043									S**	T	S												S**	Q					A						-						R**													

- *: position is not in the TEM mutation table.
- **: The novel substitution is at known position (in the TEM mutation table).
- ***: Protein sequence contains a new mutation profile in which all amino acid exchanges occur at positions not in the TEM mutation table

Table S5: New mutation profiles derived from artificial sources

Position	25*	26*	27*	28	29*	32*	36*	43*	59*	60*	66*	78*	79*	80	82*	84	86*	95*	96*	104	105*	128*	140*	152*	164	178*	179	183	184	187*	211*	212*	219*	237	240	250*	257*	258*	271*	273*	278*	284
GI	A	H	P	E	T	K	A	R	S	F	F	G	A	V	S	V	A	I	H	E	Y	T	T	L	R	R	D	P	A	A	M	E	P	A	E	L	P	S	T	D	Q	A
37778088																I	V												V													
4732115																I	P												V													
16973670										L						I							A						V				S									
3328184																I													V										A			
18996298																I													V		L	L										
2293126																I											H**		V													
74054892																I													V													H**
74054894																I													V													L**
74054890																I													V											E		L**
129278849											S																		V													
224611742																I													V												L	
183584860									G																S									T	K							
84570489												D	T	L**		I										S			V	P							R					
1003002																I								F					V													
4884789																I			L										V	P												
454853																												L**	Y**													
2995959																I																										
15081590	V	R																											V							V						
4732037												H	T	L**		I		A	R										V	P												
208244***																															L											
105958774																D**																										
116282558																I						S		F					V													
2589218																													P**													
13492214												H	T	L**		I		A	R							S			V	P												
55669167								L								I													V													
6457305			F	D	L	F	C								F	I													V													
1840129																				D**	A																					
4731623																I													V	P												
595783																													T**													
53987101																I													V									F				
146743440																I					A								V													

- *: position is not in the TEM mutation table.

- **: The novel substitution is at known position (in the TEM mutation table).

- ***: Protein sequence contains a new mutation profile in which all amino acid exchanges occur at positions not in the TEM mutation table

Table S6: Novel substitutions in lactamases from uncultured soil bacteria

GI	Amio acid at position																																						
	11	12	15	16	17	19	20	21	24	26	31	33	34	35	37	58	60	63	65	69	70	71	72	73	77	82	84	95	96	97	98	101	102	104	106	107	111	113	117	121
TEM-1	A	L	F	F	A	F	C	L	F	H	V	V	K	D	E	E	F	E	R	M	S	T	F	K	C	S	V	I	H	Y	S	D	L	E	S	P	K	L	M	E
146260002																											I													
146259995		I																									I													
146259997	R																																							
146260070																											I								A					
146260157																											I													
146260176					V																						I													
146260022																											I							K						
146260092																											I													
146260130																											I													
146260119																											I													
146260184																											I												V	
146260174																											I													
146260172																											I													
146260232																						A					I													
146260059																											I				P									
146260162																											I													
146260086																											I		Y											
146260127																											I													
146260117																											I										M			
146260054																											I													
146260028																											I								L					
146260209																											I													
146260153																											I													
146260043														G													I													
146260080																											I													
146260192																											I													
146260160				L																							I													
146260190																											I													
146259999																											I													
146260105													R														I													
146260169																K											I				P									
146260061																			H								I													
146260094																											I			C										
146260199																											I													
146260230											A																I													
146260134																											I													
146260195							Y																				I													
146260017												A	N														I													
146260122																					G		L				I													
146260101																								R			I											P		
146260211																	L										I									S				
146260007		R						F																																
146260151																											I						M							G
146260182																				V					R		I													
146260220	G																									F	I													
146260186									L																		I								P					
146260240																											I		R											
146260089			S												G			K									I													
146260207																											I													
146260068																											I													
146260180						S			L																		I								P					
146260032				S																							I													
146260045				S																							I													
146260227																											I	T												
146260009										L																	I					G								
146260202																									R		I													
146260138																G											I			C										

GI	Amio acid at position																																						
	133	136	138	140	141	151	154	159	164	175	176	177	178	179	184	189	190	192	193	199	202	206	210	211	218	220	222	228	230	236	240	243	248	252	255	259	260	262	282	284
TEM-1	T	N	L	T	T	F	N	V	R	N	D	E	R	D	A	T	L	K	L	L	A	Q	W	M	G	L	R	G	F	G	E	S	A	P	G	R	I	V	I	A
146260002															V																									
146259995															V																									
146259997															V																									
146260070															V																									
146260157															V																									P
146260176															V																									
146260022															V																									
146260092															V									I																
146260130															V							R																		
146260119				A											V																									
146260184															V																									
146260174															V									V																
146260172															V	A																								
146260232															V																									
146260059															V																									
146260162															V																					H				
146260086															V																									
146260127															V																				S					
146260117															V																									
146260054							S								V																									
146260028															V																									
146260209															V											H														
146260153			P												V																									
146260043															V																									
146260080											G				V																									
146260192															V																								R	
146260160															V																									
146260190						L									V																									
146259999															V																									
146260105															V																	P								
146260169															V																									
146260061															V																K									
146260094															V																									
146260199															V													S												
146260230															V																							A		
146260134	A												H		V																									
146260195															V																									
146260017															V																						F			
146260122															V		M																							
146260101															V																									
146260211															V																									
146260007															V					P																				
146260151															V										E															
146260182															V																									
146260220					A							G			V																									
146260186								A							V																									
146260240															V																		T		S					
146260089															V																									
146260207															V			R												E									R	
146260068														G	V												C		L											
146260180															V																									
146260032										I					V				P																					
146260045										I					V				P																					
146260227		D		A											V								R																	
146260009									H						V																			L						
146260202															V																							I		
146260138															V						T																			
